# Microparticles of High Entropy Alloys Made by Laser-Induced Forward Transfer

**DOI:** 10.3390/ma15228063

**Published:** 2022-11-15

**Authors:** Molong Han, Ashok Meghwal, Soon Hock Ng, Daniel Smith, Haoran Mu, Tomas Katkus, De Ming Zhu, Reiza Mukhlis, Jitraporn Vongsvivut, Christopher C. Berndt, Andrew S. M. Ang, Saulius Juodkazis

**Affiliations:** 1 Optical Sciences Centre and ARC Training Centre in Surface Engineering for Advanced Materials (SEAM), School of Science, Swinburne University of Technology, Hawthorn, VIC 3122, Australia; 2 Australian Research Council (ARC) Industrial Transformation Training Centre on Surface Engineering for Advanced Materials (SEAM), Swinburne University of Technology, Hawthorn, VIC 3122, Australia; 3 Academic Operations Unit, Swinburne University of Technology, Hawthorn, VIC 3122, Australia; 4 ANSTO-Australian Synchrotron, Infrared Microspectroscopy (IRM) Beamline, 800 Blackburn Road, Clayton, VIC 3168, Australia; 5 WRH Program International Research Frontiers Initiative (IRFI) Tokyo Institute of Technology, Nagatsuta-cho, Midori-ku, Yokohama 226-8503, Kanagawa, Japan

**Keywords:** laser-induced forward transfer, high-entropy alloys, microparticles

## Abstract

The controlled deposition of CoCrFeNiMo${}_{0.2}$ high-entropy alloy (HEA) microparticles was achieved by using laser-induced forward transfer (LIFT). Ultra-short laser pulses of 230 fs of 515 nm wavelength were tightly focused into ∼2.4 μm focal spots on the ∼50-nm thick plasma-sputtered films of CoCrFeNiMo${}_{0.2}$. The morphology of HEA microparticles can be controlled at different fluences. The HEA films were transferred onto glass substrates by magnetron sputtering in a vacuum ($10^{-8}$ atm) from the thermal spray-coated substrates. The absorption coefficient of CoCrFeNiMo${}_{0.2}$$\alpha \approx 6\times 10^{5}$ cm${}^{-1}$ was determined at 600-nm wavelength. The real and imaginary parts of the refractive index (n+iκ) of HEA were determined from reflectance and transmittance by using nanofilms.

## 1. Introduction

There is a growing interest in nanostructure studies of nanofilms and nanoparticles in different forms (powder and suspension in liquid). Among them, alloy nanoparticles are especially in the spotlight of nanoscience due to their unique optical and magnetic properties and applications in catalysis, biomedical engineering, and magnetic resonance imaging. High-entropy alloys (HEAs) [1], as a novel class of materials, have attracted significant attention due to the unique alloy design strategy evolving to random solid-solution phase structure, free from any intermetallic compounds [2,3]. Usually, five or more elements including transitional 3d-block and refractory metals are used for the alloy where Mo is found to reduce their propensity toward formation of intermetallic compounds [4]. The formation of simple solid solutions grants desirable material properties, such as high thermal stability, a combination of strength and ductility, and wear and corrosion resistance [5,6,7]. In addition, HEAs have also exhibited an increased damage resistance to laser irradiation, owing to the effects generated by the rise in configurational entropy, lattice distortion, and compositional complexity [8,9,10]. Nanoparticles produced by laser ablation as a simple and effective method hold many advantages over chemically produced nanoparticles. Depositing nanomicroparticles by laser-induced forward transfer (LIFT) allows patterning over large surfaces and deposition of different materials from different carrier substrates. Among recent LIFT demonstrations are the transfer of bio-materials with low damage of DNA [11] and the contact deposition for solar cells [12]. Such a particle-on-demand fabrication method can be useful for (photo/electro)-catalysis. The size of nanoparticles made by LIFT are controlled by the diameter of the laser focal spot, its fluence (J/cm${}^{2}$) and thickness of the donor film. Typically, nanofilms of 10–100 nm thickness are used. Nanofilms of any material can be sputtered or e-beam-evaporated into nanolayers, with mixtures formed by varying the number and thickness of multiple layers, for engineering of optical properties (permittivity), as we have shown for plasmonic Olympic Au–Ag–Cu metals [13].

The complex refractive index (n+iκ) of the HEA nanofilms has to be determined because it solely defines the light-matter characteristics, apart from surface roughness. By measuring the reflectance and transmittance over a wide visible-to-IR spectrum and knowing the thickness of the films, a numerical method of successive approximations can be adopted to determine the complex refractive index of a composite that takes into account the multi-reflections occurring at interface of the target film and the transparent substrate.

The non equi-atomic CoCrFeNiMo${}_{0.2}$ HEA shows strong corrosion resistance and stability at ambient temperature. The HEA coatings produced by using thermal spray processes are prone to oxidation due to in-flight oxidation. It was found that microparticles smaller than 5 μm in size become oxidised whereas those larger are only oxidised on the surface in the microfilm coatings [14]. Strong oxidation ∼50% by atomic numbers was also found in plasma-sprayed HEA [15]. However, during the processes of laser ablation and particle formation, changes in temperature might cause precipitation into other phases. Oxidation is also expected to occur. Therefore, the nanoparticles produced are expected to be comprised of multiphase intermetallic compounds (mainly the multiphase of spinel ferrite nanoparticles) instead of single-phase pure HEA. The structure, morphology, particles size distribution, and chemical composition of the produced nanoparticles is of paramount importance and has to be studied by X-ray diffraction (XRD), scanning electron microscopy (SEM), energy-dispersive spectroscopy (EDS), and Fourier transform (FTIR) and Raman spectroscopies. Additionally, there is a need for controlled deposition of nano/micro-particles of HEAs, which inherit the properties associated with HEA phases without any degradation induced by the LIFT process.

Here, we use LIFT with ultra-short laser pulses which results in the least thermal damage to donor layers due to fast thermal quenching of the transferred material/microparticle. Optical and structural analysis of nanofilms of HEA and their chemical and elemental composition was examined. Optical properties of thin sputtered nanofilms of HEA were examined.

## 2. Samples and Methods

**Figure 1:**
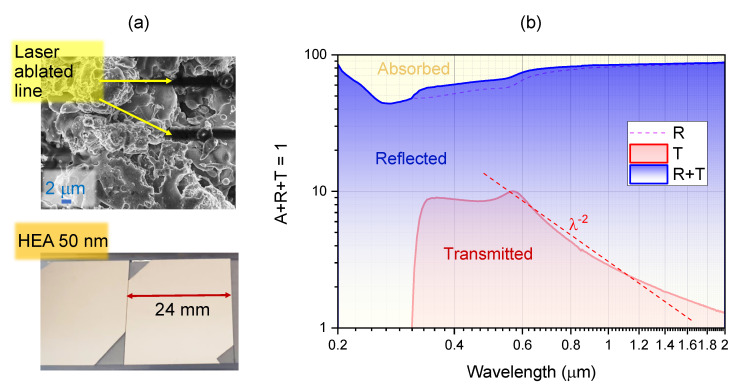
(**a**) SEM image of CoCrFeNiMo${}_{0.2}$ HEA surface (∼100 μm film on Cu-backing plate) with laser ablation trenches. The laser focus was ∼1.22λ/NA≈2.4μm for λ=515 nm laser wavelength and NA=0.26 objective lens. Photos of HTA ∼50 nm film sputtered on cover glass No. 4 ∼0.5 mm (Matsunami). Magnetron sputtering was carried at an RF bias power of 100 W rate, 7.5 nm/min deposition rate, and $10^{-5}$ atmospheric pressure (Axxis, JKLesker). (**b**) Color-coded portions of absorbed, *A*, transmitted, *T*, and reflected, *R*, light through the 50-nm-thick sputtered CoCrFeNiMo${}_{0.2}$ HEA film. R,T were directly measured by using Spectrometer (Lambda 1050 UV/VIS/NIR, PerkinElmer); note the logarithmic ordinates scale.

**High-entropy alloy.** Commercially available gas-atomized (GA) CoCrFeNiMo${}_{0.2}$ HEA powder (Jiangsu Vilory, Xuzhou, China) with a particle size og between 15–53 μm was used as the feedstock to fabricate the HEA coating onto a 3-mm-thick Cu plasma-sputtering target backing plate. The CoCrFeNiMo${}_{0.2}$ powder was sprayed by using a high velocity oxygen fuel (HVOF) thermal spray system (GTV HVOF K2, GTV Verschleißschutz GmbH, Luckenbach, Germany). The spray parameters were as follows. Kerosene fuel had a flow rate of 28 L/min. The oxygen flow rate was 950 L/min. The stand-off distance was 380 mm, and the powder feed rate was 50 g/min. The carrier gas was argon at a flow rate of 7 L/min. The surface of the HEA sputtering targets had a roughness of 1–10 μm (see SEM image in Figure 1a).

**Structural and optical characterisation.** Scanning electron microscopy (SEM) was used for structural characterisation of samples processed by laser and plasma treatments. Both a 150TWO (Raith) electron beam lithography system used in field-emission SEM mode, and a Supra 40VP (Zeiss) equipped with X-ray energy dispersion spectroscopy (EDS) unit (INCA X-act, Oxford instruments Inc., Abingdon, UK) for elemental analysis were used.

**Laser-induced forward transfer.** A femtosecond (fs-) laser microfabrication setup based on Pharos (Light Conversion) fs-laser was integrated with scanning Aerotech xy-stages and software control of laser radiation and scanning conditions (Workshop of Photonics). The LIFT mode of microparticle fabrication was used with HEA-coated glass substrates over the air gap of 10–15 μm, determined by using imaging with optical microscope. The laser beam was focused through the glass substrate and onto the interface between the HEA film and transparent donor substrate, where the 50-nm HEA film was coated onto the transparent carrier by magnetron sputtering. The transferred HEA droplets were collected by receiving substrates (Si wafer) for further analysis. The laser pulse energy $E_{p}$ was measured after the objective lens and was used to calculate fluence $F_{p}=E_{p}/\left(\pi r^{2}\right)$, where *r* is the beam waist at the focus. To achieve HEA microparticles of different morphologies, the laser fluences were varied from 5.6 J/cm${}^{2}$ to 8.9 J/cm${}^{2}$ in approximately 0.5 J/cm${}^{2}$ increments.

The X-ray photoelectron spectroscopy (XPS) analysis was performed with a Kratos AXIS NOVA spectrometer (Kratos Analytical Ltd., Manchester, UK) by using a monochromatized Al-Kα X-ray source (1486.6 eV) operating at a power of 150 W. Survey and high-resolution spectra were acquired at 160 and 20 eV pass energies, respectively. At least two spots on each sample surface with an elliptical area of approximately 0.3×0.7 mm${}^{2}$ were analyzed. A charge-neutralisation system was employed for effective charge compensation. The vacuum pressure for the XPS analysis was maintained in the order of $10^{-9}$ Torr. Data analysis and quantification were executed by using CASAXPS processing software version 2.3.17 PR 1.1 (Casa Software, Ltd., Teignmouth, UK). The atomic concentrations of each detected element were quantified based on the integral peak intensities and the sensitivity factors provided by the manufacturer. XPS analysis pertains to the thin ∼10 nm surface bound layer while EDS was contributions from ∼1 μm depths.

Magnetron sputtering was carried out on an AXXIS physical vapour deposition system (Kurt. J. Lesker). The HEA films with thickness of 14 nm, 65 nm, and 100 nm were sputtered onto the 25 mm${}^{2}$ cover glass. The glass substrates were sonicated in acetone and isopropanol alcohol for 10 min each. The power applied to the HEA target was set at 100 W and chamber pressure was $2\times 10^{-3}$ Torr, defining the deposition rate of 8.3 nm/min. Before deposition, the pressure of the deposition chamber was maintained at a high-vacuum (∼$10^{-8}$ Torr) to reduce the effect of oxygen.

The optical characterisation of nanofilms of HEA was carried out with a Lambda 1050 UV/VIS/NIR spectrometer (PerkinElmer), Waltham, MA, USA. and a UV-1601 (Shimadzu), Kyoto, Japan. A tungsten halogen lamp was used as the main light source, complemented with a deuterium lamp for shorter wavelengths. A PbS detector and a photomultiplier tube were equipped to detect near-infrared and UV/VIS (860 nm and shorter) wavelengths, respectively. Both reflectance *R* and transmittance *T* were measured for the three HEA nanofilms of different thickness (thickness: 100 nm, 65 nm and 14 nm) over spectra from 175 nm to 2000 nm with a high scanning resolution of 1 nm/scan. The thickness of films was determined by using a white light profilometer (ContourGT, Bruker), Billerica, MA, USA.

**Numerical modeling.** A three-dimensional (3D) finite-difference time-domain (FDTD) simulation was performed by using commercial software (Lumerical FDTD 2022 R1, Ansys, Inc.). The snowman-like microparticle shape was simplified as a combination of a bottom sphere (diameter: 500 nm) and a top spheroid (semi-major diameter: 500 nm; semi-minor diameter: 250 nm). The HEA material was defined by the calculated complex refractive index (n+iκ) over the spectrum range from 175 nm to 2000 nm.

## 3. Results and Discussion

Non equi-atomic CoCrFeNiMo${}_{0.2}$ high-entropy alloy (HEA) nanofilms were prepared by physical vapor deposition (PVD) in a vacuum from ∼100 μm coatings made on the plasma-sputtering targets by the HVOF process (Section 2). Then, HEA nano-/microparticles were made by LIFT by using ablation of HEA nanofilms irradiated by single femtosecond laser pulses.

### 3.1. Optical Characterisation of HEA Nanofilms: (n,κ)

**Figure 2:**
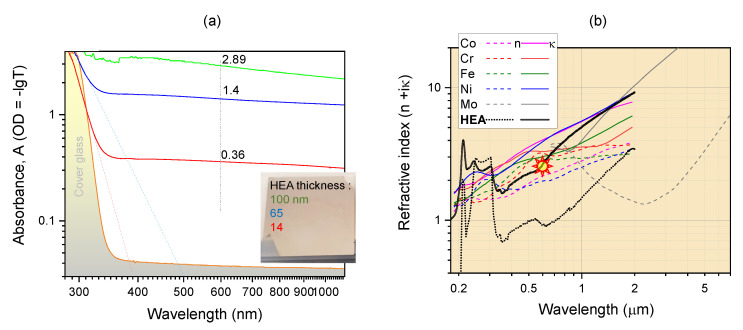
(**a**) Absorption spectra of CoCrFeNiMo${}_{0.2}$ films of different thickness; cover glass absorption (shaded profile) was compensated by using reference (Shimadzu). (**b**) Real and imaginary parts of the refractive index (n+iκ) of the constituent metals and HEA. Marker shows the point of κ=2.685 at λ=600 nm of the CoCrFeNiMo${}_{0.2}$ HEA; absorption coefficient $\alpha =4\pi \kappa /\lambda \approx 6\times 10^{5}$ cm${}^{-1}$ is obtained from the Beer–Lambert law.

By measuring *R* and *T* for an HEA film on a known thickness (strongly absorbing) on a transparent substrate, the complex refractive index (n+iκ) of the HEA nanofilms can be determined [16] by using the following equation used for Pd–Au alloys [17]:

(1) $$R=\frac{{\left(1-n_{1}\right)}^{2}+\kappa_{1}^{2}}{{\left(1+n_{1}\right)}^{2}+\kappa_{1}^{2}};\;\;\;\;T=\frac{16n_{2}\left(n_{1}^{2}+\kappa_{1}^{2}\right)}{\left[{\left(n_{1}+n_{2}\right)}^{2}+{\left(\kappa_{1}+\kappa_{2}\right)}^{2}\right]\left[{\left(1+n_{1}\right)}^{2}+\kappa_{1}^{2}\right]}e^{\frac{-4\pi d}{\lambda }},$$

where the $n_{1},\kappa_{1},n_{2}$, and $\kappa_{2}$ are the real and imaginary parts of the complex refractive index of HEA film and cover glass, respectively, *R* and *T* are the overall reflectance and transmittance at the normal incidence, *d* is the thickness of the HEA film, and the λ is the wavelength. Figure 1b shows experimentally measured *R* and *T* presented as fractions of transmitted, reflected, and absorbed portions of incidental light. This was used for the calculation of (n,κ) by using Equation (1) with thickness *d* determined by optical profilometer. Figure 2 shows Beer–Lambert absorbance of different thickness of HEA films on cover glass (a) and (n,κ) data for the constituent metals and the HEA film (from the data shown in Figure 2b). Interestingly, a sub n<1 refractive index was observed at visible wavelengths. This is promising for engineering of epsilon-near-zero (ENZ) materials with permittivity (“epsilon”) $\varepsilon ={\left(n+i\kappa \right)}^{2}$, which are used in perfect absorbers [18]. The free-carrier absorption can be recognised by $\kappa \propto \lambda^{2}$ for λ>0.6μm.

### 3.2. Microparticles and Their Arrays out of HEA

HEA films made by thermal spray are microrough (Figure 1a) and were plasma-sputtered into 50 nm films on glass substrates for characterisation as well as for LIFT. Figure 1b shows color-coded portions of absorbed *A*, transmitted *T*, and reflected *R* light comprising the energy conservation by A+R+T=100% (note the log–log scale of the spectrum). Only ∼10% of light is transmitted over the visible part of spectrum 400–800 nm. This is typical for metal coatings of $1/e^{2}$-level of transmitted power/intensity. The skin depth 1/α is tens of nm for the absorption coefficient α=4πκ/λ. From approximately λ=600 nm, transmittance drops at longer wavelengths. This is typical for free carrier absorption, which increases as $\propto \lambda^{2}$ (see dashed line with slope of −2). The portion of reflected light increases with λ, whereas transmission decreases for the same reason—the high free carrier density. This defines a smaller portion of light absorbed at the IR spectral window where the 50-nm HEA film becomes negligibly thin.

The absorption coefficient α was determined from direct measurement of transmittance by using an uncoated glass reference (No. 4 Matsunami cover glass) in the reference arm of the spectrometer (Shimazu) for different thicknesses of HEA coating; the glass substrate contribution is canceled in the plotted spectra (Figure 2a). The thickness of deposited HEA film was calibrated by using an optical profilometer; a 7.5-nm/min deposition rate of film was used. The extinction coefficient of the HEA film was κ=2.685, which yields $\alpha =6\times 10^{5}$ cm${}^{-1}$ absorption coefficient (at the 600 nm wavelength selected). This value is consistent with the extinction of the metals used in the alloy (Figure 2b).

The established strong absorption of HEA nanofilms (Figure 2) is defined by the optical density OD≈1 with transmitted intensity $I_{t}=I_{in}10^{-OD}\equiv e^{-\alpha d}$ for the thickness *d*. For fs-laser LIFT, we used a wavelength of 515 nm, which is in this strongly absorbing range. The energy of the laser pulse is deposited into the skin depth, which closely matches the thickness of the HEA film. The forward momentum of laser pulse is cumulative of single-photon momenta p=h/λ, where *h* is the Plank’s constant, and is deposited by absorption. The laser beam energy is linked to the momentum via E=pc, and *c* is the speed of light. In case of reflection, double the large momentum is deposited due to the change in direction of light upon reflection. The melted and pushed droplet from the carrier layer is transferred onto the receiving substrate.

**Figure 3:**
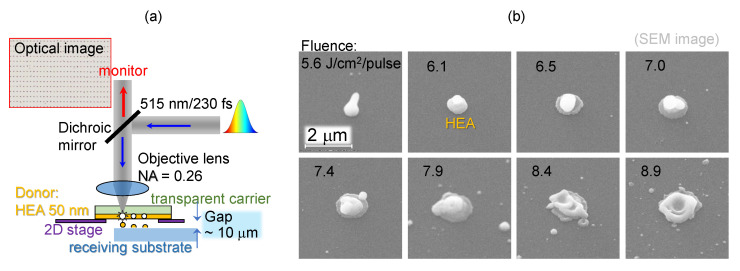
(**a**) LIFT of HEA film. (**b**) SEM images of single sub-1 μm particles of CoCrFeNiMo${}_{0.2}$ transferred on a cover glass at different fluences per pulse (J/cm${}^{2}$/pulse) under NA=0.26 focusing. Laser irradiation: 230 fs, 515 nm. Thickness of HEA was 50 nm.

We used a spacing of ∼10 μm between the carrier and receiver as was judged from observation in an optical microscope (Figure 3a; height control ∼1 μm). A fairly narrow fabrication window of fluence 5–6 J/cm${}^{2}$/pulse exists for LIFT for the most uniform particles. When the laser fluence was just above the ablation threshold, droplet-shape microparticles with a size smaller than the laser spot size were transferred by LIFT. As the laser fluence increased, the shape of the microparticles evolve from being snowman-like to multilayer pancake-like. At high fluences, the transferred microparticles were approximately the same as the laser spot size with much debris in the surrounding area (Figure 3b).

**Figure 4:**
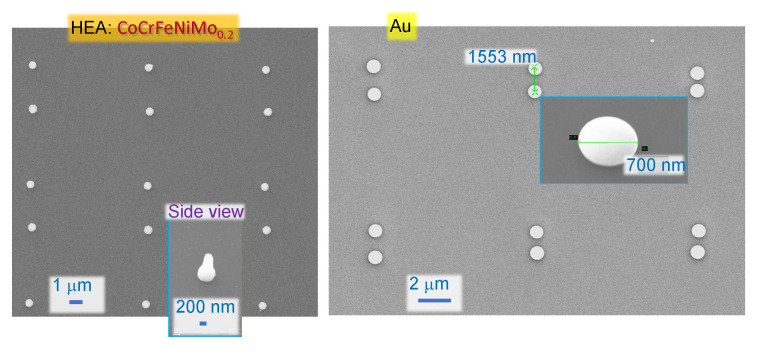
Arrays of microparticles made by LIFT of high CoCrFeNiMo${}_{0.2}$ and Au transferred onto Si wafer at pulse energies $E_{p}=344$ nJ or 5.6 J/cm${}^{2}$ (on sample) for HEA and 816 nJ or 14 J/cm${}^{2}$ for Au under NA=0.26 focusing. Laser irradiation: 230 fs, 515 nm. Thickness of HEA and Au was 50 nm.

With the proper choice of pule energy/fluence, it was possible to transfer uniform HEA sub-1 μm particles onto any receiving substrate. The pulse fluence of 5.6 J/cm${}^{2}$, for example, allows droplet-shape HEA micro-particles to be transferred in a uniform pattern. As shown in Figure 4, HEA microparticles were printed in a paired-particle array by LIFT (particle pair distance: 3 μm; array period: 10 μm). For comparison, ellipsoid-shape gold microparticles were achieved by LIFT from a 50 μm gold film with a higher pulse fluence of 14 J/cm${}^{2}$; however, for HEA such irradiance would cause loss of shape and size.

### 3.3. Material Characterisation

**Figure 5:**
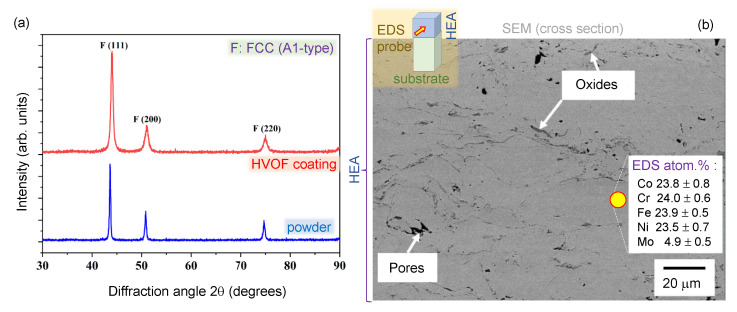
Thick HEA film deposited by high-temperature spray. (**a**) X-ray diffraction (XRD) of diffractograms of gas atomized CoCrFeNiMo${}_{0.2}$ powder and HVOF sprayed coating (GTV HVOF K2, GTV Verschleißschutz GmbH, Germany). (**b**) The cross-sectional scanning electron microscopy (SEM) image of HVOF CoCrFeNiMo${}_{0.2}$ coating along with EDS analysis of the coating phase in atomic percentage.

The XRD patterns for a gas-atomized CoCrFeNiMo${}_{0.2}$ powder and HVOF CoCrFeNiMo${}_{0.2}$ coating are illustrated in Figure 5a. The phase composition of the powder is primarily a single solid solution based on face-centered cubic (FCC) phase structure, which was retained in the coating produced by using HVOF without indicating any peaks relating to oxides or intermetallic compounds. The higher magnification back-scattered electron (BSE) SEM micrograph of HVOF coating shown in Figure 5b revealed intersplat regions and randomly dispersed pores without any substantial depth variation within the microstructure of the coating. In addition, minor oxide fragments across the intersplat regions were identified with an average chemical composition of 25.2 at% O, 17.2 at% Co, 18.7 at% Cr, 19.4 at% Fe, 15.9 at% Ni and 3.6 at% Mo when measured by using point EDS analysis. Therefore, the composition of the oxides is attributed to the spinel-based oxide structure, which was developed due to the in-flight oxidation characteristics of the HVOF process. More importantly, the absence of oxide peaks in the XRD diffractogram of the CoCrFeNiMo${}_{0.2}$ coating signified their relatively low concentration across the microstructure. The oxygen-rich regions were only observed by XPS, which is surface sensitive and is discussed below.

With several processing steps involved from the thermal spray, Ar-ion plasma sputtering to microparticle formation by laser ablation, it is important to establish chemical changes. Laser ablation is taking place with electron excitation and departure from the surface. In chemistry terms, this is oxidation (loss of an electron). Ar-ion sputtering of HEA films, which are composed of squashed micro-droplets of tens of μm can have a preferential sputtering of the interfaces and boundaries. The fs-laser ablation and LIFT is a process and formation of different phases of materials defined by nonequilibrium in which highly dynamic ion separation governed by difference in the ion masses [19,20] is possible.

**Figure 6:**
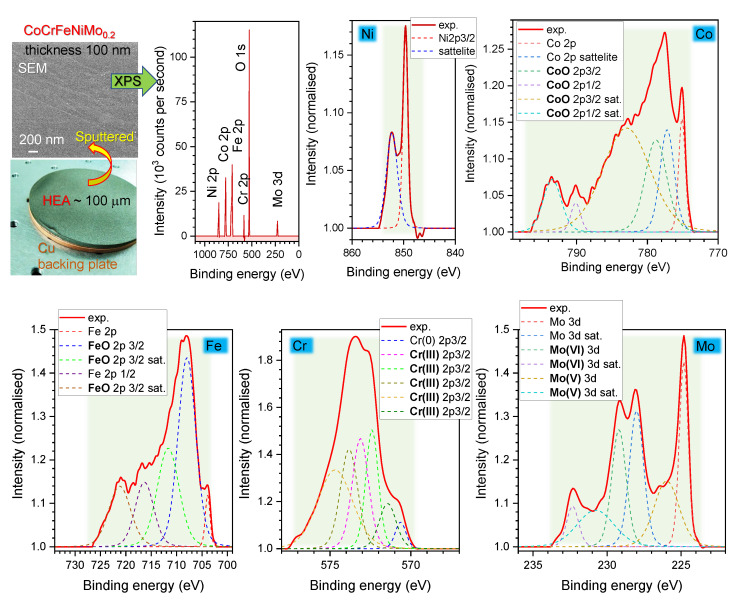
X-ray photoelectron spectroscopy (XPS) of CoCrFeNiMo${}_{0.2}$ 100-nm-thick film transferred on a cover glass by magnetron sputtering. Such a film was used as as a carrier for LIFT. Magnetron sputtering was carried out at the order of $10^{-3}$ Torr. The CasaXPS software package was used for XPS data analysis. A wide-range binding energy spectrum and detailed elemental bands (normalised) are shown; background highlights (green) show the energy window used for quantification. Bold markers in the legends are for the oxide states: CoO, FeO, Cr(III) [chromia Cr${}_{2}$O${}_{3}$], Mo(VI) [MoO${}_{3}$], and Mo(V) [Mo${}_{2}$O${}_{5}$].

To characterise the target source used for the LIFT printing of single microdroplets of HEA, XPS analysis of 100-nm-thick sputtered films was carried out (Figure 6). The presence of oxides was most pronounced for CoO, FeO, chromia Cr_2_O_3_, and MoO_3_; oxides are shown on the higher binding energy side (Figure 6). Qualitatively similar results for HEA prepared by thermal and plasma spray were observed [14,15]. High-vacuum ($10^{-8}$ Torr) sputtering of HEA targets onto glass was not expected to contribute to a larger portion of oxides in the film. However, some surface oxidation of the HEA nanofilm on glass is expected at room conditions after deposition, e.g., this was observed for Cr coating. It is noteworthy that the oxide surface layer is on the far side of the glass–HEA film interface which is irradiated for LIFT. Hence, the fs-laser pulse interacts mostly with the less oxidised part of the HEA film.

Because HEA targets were made by using thermal spray, there was carbon presence in the film as well. The ratio of O 1s to C 1s peaks was 43.38:38.21% and 42.08:38.77% in two different samples. The ratio of oxygen to all the metals (Ni, Co, Fe, Cr, Mo) was: 70.21% to (3.19-Ni; 7.91-Co; 11.18-Fe; 5.63-Cr; 1.88%-Mo) and 68.72% to (3.97-Ni; 7.69-Co; 12.12-Fe; 5.57-Cr; 1.91%-Mo) in the very same samples/areas (Figure 6). Composition differences were not expected between those two areas/samples and are caused solely by uncertainties. If carbon is added for 100% of the composition of the samples, one would find the following atomic ratios [%]: 43.38(O), 38.21(C) and for metals 1.97-Ni, 4.89-Co, 6.91-Fe, 3.48-Cr, 1.16-Mo (sample No. 1); 42.08(O), 38.77(c) and 2.43-Ni, 4.71-Co, 7.42-Fe, 3.41-Cr, 1.17-Mo (sample No. 2).

**Figure 7:**
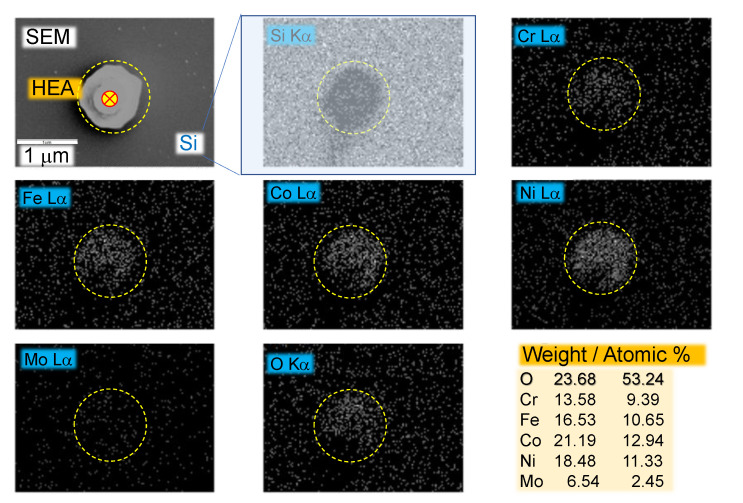
X-ray energy-dispersive spectroscopy (EDS) mapping of a microparticle of CoCrFeNiMo${}_{0.2}$ on Si substrate (Oxford on Zeiss SEM microscope).

The elemental composition of single microparticles of HEA printed by LIFT was characterised by using EDS (Figure 7). Both XPS and EDS results indicate that Cr, Fe, Co, Ni, and Mo are evenly distributed on the sputtered nanofilm. LIFT was carried out onto a Si substrate and oxygen contribution from the substrate’s native oxide (∼2 nm) is minimal, because EDS probes the deep subsurface volume at ∼1 μm depth, in contrast to the surface bound (nanometres) XPS. Oxidation of microdroplets (on Si) was smaller as compared with XPS data of the nanofilm used for laser printing (Figure 6) judging from the O-atomic content of selected microparticles reaching ∼53% atomic (here, C was not used for the quantification and it was negligible in the spectral survey, most probably due to oxidation during LIFT). This is consistent with XPS data for the HEA film on glass, which was used as a source for droplet transfer. The reference EDS results of sub-mm HEA film measured into the film (side view SEM) is shown in Figure 5 together with X-ray diffraction results.

**Figure A3:**
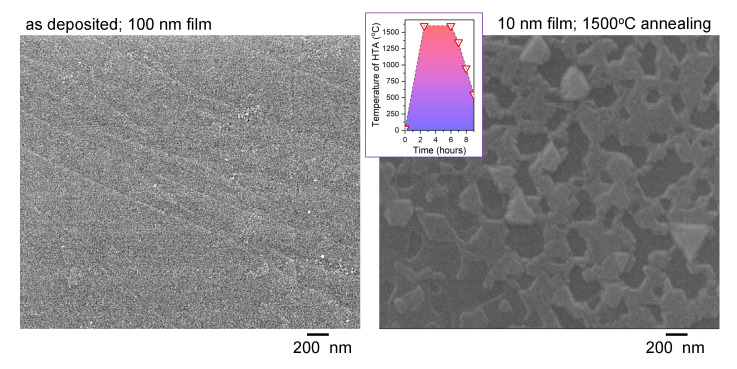
SEM images of the as sputtered CoCrFeNiMo${}_{0.2}$ film and after high-temperature annealing at 1500 ${}^{\circ }$C for 2 h under N${}_{2}$ flow to avoid oxidation. The substrate was Al${}_{2}$O${}_{3}$ crystalline substrate with c-plane used for deposition of HEA (sapphire melts at ∼2100 ${}^{\circ }$C). For SEM imaging of the annealed sample 10 nm of Cr was coated to reduce charging.

The comparative EDS analysis between target coating and particle revealed significant differences with the chemical composition. The oxygen content within the particle increases significantly as compared to the target due to the high temperature involved in the LIFT process. The substantial decrease in elemental content of Co, Cr, Fe, and Ni attributed to the equatorial proneness of these elements toward the oxidation. By using high-temperature melting substrates such as crystalline Al2O3, it is possible to prepare HEA donor films for LIFT (Figure A3). Moreover, thermal remelting of sputtered HEA can be used to produce alloys of better crystalline quality. Even a strategy to sputter 2–5 nm films of separate metals on a preheated substrate or to anneal films afterward is another avenue for the production of HEA and needs to be investigated since it was found to work for the Pd–Au alloy [17].

## 4. Conclusions and Outlook

It was shown that films of tens of nm thickness had optical properties closely matching those of metals with absorption coefficient corresponding to the strong absorbance $\alpha \approx 5\times 10^{5}$ cm${}^{-1}$ in the visible spectral range. Strong reflectance in the near-IR region is also consistent with free-carrier (electron) absorption $\alpha \propto \kappa \propto \lambda^{2}$ at longer wavelengths (as expected for metals). Direct laser writing of single sub-1 μm particles and their arrays was demonstrated in a single pulse mode for focusing into a ∼2.4 μm spot.

Future studies of HEA oxidation during LIFT can be carried out by using synchrotron IR microspectroscopy because metal oxides have absorption bands over the fingerprinting IR region. Also, formation of metallic glass phases is expected due to fast thermal quenching by using fs-laser LIFT. High-resolution X-ray and neutron-scattering techniques are promising for the revelation of further details on composition and morphology.

## Figures and Tables

**Figure A1:**
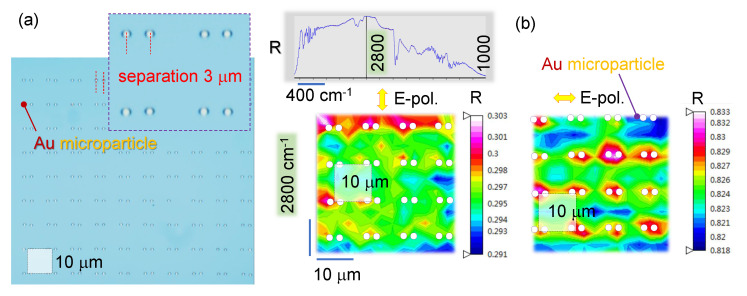
(**a**) Optical image (reflection) of Au microparticles on Si substrate. (**b**) Reflection spectral mapping of Au microparticles (pairs) patterned at $\Lambda_{x,y}=10\;\mu$m array at two perpendicular polarisations; map taken at 2800 cm${}^{-1}$ or 3.57 μm or 83.94 THz. Circular markers are locations of Au microparticles of ∼0.7 μm. Au microparticles were LIFT transferred from 50 nm film by using NA=0.26 objective lens at pulse energy $E_{p}=816$ nJ. Separation between Au microparticles 3 μm. The light source is Australian Synchrotron. The mapping was carried out by MCT detector with a scanning pinhole aperture of 3 μm. The reference was flat Si substrate.

**Figure A2:**
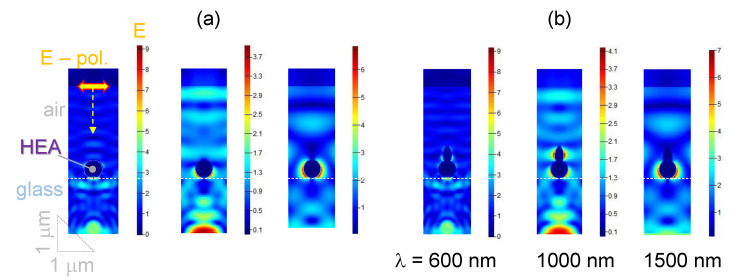
Light field enhancement by a HEA microparticle (on glass) at different wavelengths calculated by FDTD for spherical (**a**) and snowman (**b**) structures as observed in experiments (Figure 3b; incident E-field is |E|=1. HEA is modeled as homogeneous refractive index (n+iκ) of HEA (Figure 2b). Boundary conditions: perfect matching layer.

## Data Availability

The data presented in this study are available on request from the corresponding author.

## Appendix A. Light-Field Enhancement by Au Micro-Particle at IR Wavelengths

Samples of Au microparticles were patterned on Si (Figure A1a; similar as in Figure 4) for mapping of reflectance *R* at selected wavenumbers $\tilde{\nu }$ at two perpendicular polarisations (at the IR micro-spectroscopy beamline of the Australian Synchrotron). Example of *R* map at $\tilde{\nu }=2800$ cm${}^{-1}$ (λ=3.57μm) is shown in Figure A1b. Slight light E-field enhancement is observed in reflection from regions between sub-1μm Au spheres (aligned in pairs along x-direction (a)) for the horizontal x-pol. This corresponds to the alignment direction of microparticles in the pair. Separation between two microparticles was ∼3 μm and it was sub-wavelength for the used mapping wavelength of λ=3.57μm. Stronger reflectivity is expected from Au microsphere and due to light concentration on the pair of Au microspheres.

At perpendicular (y-pol.), a small enhancement was observed at the position of the pair of Au microparticles rather in between the lines; it is noteworthy that the separation between the lines of paired Au microparticles was larger by approximately the gap between them in the x-direction. There were strong edge effects at the extremities of the read-field. Mapping was made by using a point MCT detector. Synchrotron radiation has a complex polarisation distribution due to beam extraction from the synchrotron by mirror with a gap at its center (the 1${}^{\mathrm{st}}$ fork-mirror) and direction of observation. This usually causes not even intensity across the focus which contributes to unevenness of intensity maps on 3D sturfaces.

## Appendix B. Light Enhancement by Microparticle of HEA

Light field enhancement by a single HEA microparticle: sphrere and snowman were modeled by using FDTD at different wavelengths through the visible to near-IR spectral range (Figure A2). The experimentally determined spectral dependence of n,κ was used for the modeling (Figure 2b). Expected light field localisation by metallic nanoparticle was observed with most of light concentration and enhancement taking place around a metallic nanoparticle. Such nano-/microparticles can be used for surface-enhanced Raman scattering (SERS) sensors as well as color markers [21].

The phase change of the reflected field $E_{r}$ (in air) from the surface with refractive index *n* is defined by $E_{r}=-\frac{n-1}{n+1}E_{in}$, where $E_{i}$ is the incident E-field; the minus sign means the opposite phase or the π-shift. When n>1, there is a π-shift between the incident and reflected E-fields of light while for the n<1, there is no phase change between $E_{r}$ and $E_{i}$. The latter situation can be realised at the visible spectral range where n<1 for HEA (see, λ=600 nm case in Figure A2).

## Appendix C. High Temperature Annealing of HEA

Another possible technique for fabrication of nano-/microparticles is the high temperature dewetting of nano-thin films. For example 20 nm of Au on fused silica SiO${}_{2}$ substrate can be transformed into nanoislands by 650 ${}^{\circ }$C annealing for 1 h. Thin 10-nm CoCrFeNiMo${}_{0.2}$ film was sputtered on crystalline Al${}_{2}$O${}_{3}$ substrate and annealed at high 1500 ${}^{\circ }$C temperature for possible dewetting (Figure A3). Surface morphology was obviously changed from nanosmooth initial HEA film revealing structures of 100–300 nm in lateral cross sections after HTA (high temperature annealing) (see HTA protocol in the inset of Figure A3) under SEM observation. Further studies will be carried out for structural and chemical composition of the film. Moreover, sublimation of HEA is another important phenomenon which needs to be investigated by HTA.
